# Anatomical and Patient-Reported Outcomes After Non-Ablative Er:YAG Laser Therapy for Genitourinary Syndrome of Menopause: A Prospective Real-World Cohort Study

**DOI:** 10.3390/healthcare14091180

**Published:** 2026-04-28

**Authors:** Stephanie Kauffmann, Montserrat Girabent Farrés, Cristina Naranjo Ortiz, Laia Blanco-Ratto, Manuel Del Campo Rodríguez, Inés Ramírez-García

**Affiliations:** 1 RAPbarcelona Physiotherapy Clinical Center, 08037 Barcelona, Spain; stephanie.kauffmann@rapbarcelona.com (S.K.); laia.blanco@rapbarcelona.com (L.B.-R.); 2 Doctoral Programme in Health, Wellbeing and Bioethics, Universitat Ramon Llull, 08025 Barcelona, Spain; 3 Campus Docent Sant Joan de Déu, Universitat de Vic-Universitat Central de Catalunya (UVic-UCC), 08500 Barcelona, Spain; montserrat.girabent@sjd.edu.es; 4 Yale Cancer Center, Yale School of Medicine, Yale University, New Haven, CT 06520, USA; cnaranjoortiz@gmail.com; 5 Teknon Medical Center, 08022 Barcelona, Spain; ginedelcampo@gmail.com; 6 Blanquerna School of Health Sciences, Universitat Ramon Llull, 08025 Barcelona, Spain; 7 GHenderS Research Group, Blanquerna School of Health Sciences, Universitat Ramon Llull, 08022 Barcelona, Spain

**Keywords:** Er:YAG, female sexual function, genitourinary syndrome of menopause, non-ablative Er:YAG vaginal laser therapy, quality of life

## Abstract

**Highlights:**

**What are the main findings?**

- Non-ablative Er:YAG laser therapy was associated with a significant reduction in vaginal hiatus and improvement in cystocele stage in a real-world outpatient setting.
- Quality-of-life domains related to social embarrassment and psychosocial impact improved, whereas global sexual function and urinary symptom scores showed limited short-term change.

**What are the implications of the main findings?**

- These exploratory findings provide objective and patient-reported data supporting further evaluation of non-surgical, energy-based interventions within outpatient care models for genitourinary syndrome of menopause
- The observed discrepancy between structural and functional responses indicates that additional controlled studies are needed to better define the clinical role of this intervention within outpatient care pathways.

**Abstract:**

**Background/Objectives**: This exploratory single-arm study evaluated the effects of non-ablative Erbium-doped Yttrium Aluminum Garnet (Er:YAG) laser therapy in a real-world healthcare setting. **Methods**: A prospective pre–post study was conducted in 47 postmenopausal women who received two sessions of non-ablative Er:YAG vaginal laser therapy (IncontiLase^®®^/IntimaLase^®®^). Assessments were performed at baseline and two follow-ups (FSFI, ICIQ-SF, I-QOL, and Oxford Scale). Wilcoxon signed-rank tests and Spearman correlations were used. **Results:** Vaginal hiatus was significantly reduced from 2.5 cm (IQR 2.0–3.0) to 2.0 cm (IQR 1.0–3.0) (*p* < 0.001). Vaginal length showed a small, non-significant increase, and pelvic floor strength was unchanged. Total FSFI scores remained stable; pain showed a non-significant upward trend, and arousal decreased transiently. ICIQ-SF scores did not significantly improve, although they correlated inversely with vulvar energy at second follow-up (*r* = −0.424; *p* = 0.016). I-QOL domains showed short-term improvements in social embarrassment (*p* = 0.002), psychosocial impact (*p* = 0.002), and behavioral limitations (*p* = 0.013) at first follow-up. Cystocele stage improved at second follow-up (*p* = 0.013). **Conclusions**: Non-ablative Er:YAG vaginal laser therapy was associated with reduced vaginal hiatus and produced short-term improvements in select quality-of-life domains and cystocele stage, while effects on sexual function and urinary symptoms were limited. Findings remain exploratory and inform the design of future controlled studies evaluating innovative outpatient care models for GSM. Given the absence of a control group and short follow-up, these findings should be interpreted as hypothesis-generating and may be influenced by placebo or expectation effects.

## 1. Introduction

Genitourinary syndrome of menopause (GSM), formerly known as urogenital atrophy, is a multifactorial clinical condition that affects a high percentage of women during the menopausal transition and post-menopause. It is characterized by anatomical and functional alterations of the urogenital tract associated with estrogen deficiency [1]. This syndrome encompasses vaginal symptoms such as dryness, burning, itching, irritation, dyspareunia, and postcoital bleeding, as well as urinary symptoms including urgency, increased frequency, urinary incontinence, and dysuria. Epidemiological studies estimate that 40–60% of postmenopausal women experience GSM symptoms; however, the true prevalence may be underestimated due to underreporting, normalization, and limited clinical inquiry [2].

The impact of GSM extends beyond the physical sphere, affecting emotional, psychological, sexual, and social well-being. GSM significantly impairs quality of life, self-image, and sexual function [3]. Although local estrogen therapy and other forms of hormone therapy remain among the most effective treatments for GSM, they are not appropriate for all women. Recent regulatory updates from the U.S. Food and Drug Administration (FDA) have begun the process of removing certain outdated boxed warnings regarding menopausal hormone therapy, reflecting a reassessment of its overall risk–benefit profile [4]. This ongoing need for safe and effective non-hormonal alternatives has contributed to growing interest in energy-based technologies as potential therapeutic options.

In recent years, non-ablative Er:YAG vaginal laser therapy, particularly Erbium-doped Yttrium Aluminum (Er:YAG) technologies such as IntimaLase^®®^ and IncontiLase^®®^, has gained attention as a treatment strategy for GSM. This modality is hypothesized to induce controlled photothermal stimulation, promoting collagen remodeling, extracellular matrix synthesis, and epithelial regeneration without extensive thermal damage or prolonged recovery [5], although direct links between these tissue-level effects and clinical outcomes remain incompletely established. Previous studies have reported that it improves anatomical parameters such as diameter and vaginal hiatus, alleviates symptoms of dryness, dyspareunia, and urinary incontinence, partially restores sexual function, and enhances quality of life [6,7,8,9].

Despite these preliminary findings, controversy persists due to heterogeneous methodologies, a limited number of high-quality randomized controlled trials, small sample sizes, and variable follow-up durations. Many studies evaluated isolated outcomes rather than integrated anatomical, functional, and subjective domains. Additionally, the frequent absence of control groups, reliance on subjective measures, and use of manual anatomical assessments limit internal validity and generalizability [10,11,12]. Further research using standardized, multi-domain evaluation frameworks is therefore warranted.

In this context, the present study aimed to evaluate longitudinal changes in anatomical (vaginal hiatus, vaginal length), functional (urinary incontinence, pelvic floor strength), and subjective (sexual function, quality of life) parameters in postmenopausal women with GSM, treated with non-ablative Er:YAG vaginal laser. This preliminary single-arm design provides exploratory data to inform future controlled trials. Given the exploratory nature and lack of comparator, the study was designed as hypothesis-generating rather than confirmatory.

## 2. Materials and Methods

### 2.1. Study Design and Population

A prospective single-arm, pre–post preliminary interventional study was conducted to assess the impact of non-ablative Er:YAG vaginal laser therapy on anatomical and functional outcomes in postmenopausal women diagnosed with GSM. A total of 47 postmenopausal women were recruited at a specialized medical unit. Eligible participants presented with at least one clinical manifestation of GSM such as vaginal dryness, dyspareunia, vaginal laxity, urinary incontinence, or sexual dysfunction, and provided written informed consent prior to enrollment.

The study included postmenopausal participants who were experiencing GSM symptoms and were able to comply with study procedures. Participants were excluded if they did not have GSM, had an active pelvic infection, had received prior pelvic radiation, had unhealed vaginal wounds, had used systemic estrogen therapy within the previous six months, or had not provided signed informed consent. Recruitment began with the first enrollment on 27 January 2024 and concluded with the final enrollment on 3 October 2024. The study was registered under ISRCTN52370656. Ethics committee approval was obtained from the Blanquerna CER Committee under approval number n° 2024-01-01 (24 January 2024). All participants provided written informed consent.

### 2.2. Intervention Protocol

Each subject underwent treatment using the IncontiLase^®®^ and/or IntimaLase^®®^ protocols, delivered through a non-ablative Er:YAG laser device (model SP DYNAMIS, S/N 14003339. Fotona, d.o.o., Ljubljana, Slovenia). The laser applies controlled photothermal energy to elevate epithelial temperatures to approximately 65 °C, promoting collagen remodeling and mucosal tightening. Participants received two standardized sessions spaced 6–8 weeks apart. A third session was offered at clinician discretion based on individual symptom progression and anatomical findings.

### 2.3. Data Collection and Clinical Variables

Collected variables included demographic data (age, body mass index, parity), gynecologic and obstetric history (presence and type of urinary incontinence, pelvic organ prolapse), and laser protocol details (number of sessions, energy delivered to the anterior and posterior vaginal walls, circumferential areas, and vulva), as well as both anatomical and functional outcomes. Anatomical assessments involved measuring vaginal hiatus and total vaginal length. These examinations were performed with the bladder and rectum emptied, in accordance with guidelines from the International Continence Society and the International Urogynecological Association [13]. A Sims speculum was used to retract the vaginal walls and facilitate direct visualization [14]. The vaginal hiatus was recorded as the maximal transverse distance between the vaginal borders during a Valsalva maneuver. Total vaginal length was measured from the posterior fornix to the hymenal ring, with manual repositioning of point C or D when required [15]. Pelvic floor muscle strength was evaluated through digital vaginal palpation by a trained physiotherapist using the Oxford Scale (grade 0–5). No formal intra-rater reliability assessment was conducted, which may introduce measurement variability. All measurements were performed following a standardized protocol to minimize intra-observer variability.

### 2.4. Assessment Tools and Timing of Evaluations

Participants were assessed using validated instruments evaluating sexual function, urinary symptoms, and quality of life. The Female Sexual Function Index (FSFI) assessed desire, arousal, lubrication, orgasm, satisfaction, and pain. The International Consultation on Incontinence Questionnaire—Short Form (ICIQ-SF) evaluated frequency, severity, and impact of urinary incontinence on daily activities. The Incontinence Quality of Life Questionnaire (I-QOL) assessed psychosocial burden, social embarrassment, and behavioral limitations.

Pelvic organ prolapse was assessed clinically and classified by compartment (cystocele, rectocele, uterine prolapse) according to the Pelvic Organ Prolapse Quantification (POP-Q) system (staged I–IV).

Assessments were conducted at three predefined time points:

- Baseline evaluation (EB): prior to the first laser session.
- First follow-up (FU1): 6–8 weeks after the first session.
- Second follow-up (FU2): 6–8 weeks after the second session.

All clinical evaluations were performed by gynecologists and physiotherapists trained and certified in pelvic floor rehabilitation and in the standardized use of the assessment tools applied in this study. Laser treatment sessions were conducted by a physician who was aware of the patient’s clinical information and the corresponding session number. Anatomical and functional assessments were performed by a specialized pelvic floor physiotherapist who was blinded to treatment parameters (energy and protocol), but not to treatment timing, resulting in partial blinding. The type and total energy delivered were recorded exclusively by the treating physician.

### 2.5. Statistical Analysis

Statistical analysis was carried out using IBM SPSS Statistics version 26. Given the non-normal distribution of data, the Wilcoxon signed-rank test was applied to compare paired data across time points. Effect sizes (*r*) were calculated for primary outcomes to support interpretation of clinical relevance. The correlation analysis was conducted using the Spearman correlation coefficient. Statistical significance was set at a *p*-value < 0.05. No adjustments were made for multiple comparisons, as is common in pilot/preliminary designs. No correction for multiple comparisons was applied due to the exploratory design; therefore, findings should be interpreted cautiously as potentially inflated Type I error. Mild or moderate adverse events were systematically recorded. The sample size (*n* = 47) was based on a paired pre–post design assuming a moderate effect size (Cohen’s d = 0.5), alpha level 0.05, and power of 80%. The study was powered for a primary comparison only and was not powered to detect differences across multiple endpoints. Therefore, all secondary and exploratory analyses should be interpreted cautiously. G*Power 3.1 estimated a minimum of 34 participants; therefore, the achieved sample provides adequate statistical power. Adverse events and tolerability were recorded at each visit; no serious treatment-related events were observed. Where appropriate, confidence intervals were considered to support interpretation of effect sizes and the precision of estimates.

## 3. Results

All results should be interpreted within the exploratory framework of this single-arm study.

### 3.1. Participant Characteristics

Table 1 presents the demographic and clinical characteristics of the 47 postmenopausal women included in the study. The mean age was 58.1 ± 8.99 years and the mean BMI was 23.2 ± 3.64 kg/m^2^. Most women had two full-term deliveries (52.3%), followed by those with one (25.0%) or three (15.9%) births. Urinary incontinence was reported by 46.8% of participants, while pelvic organ prolapse was present in 66.0%, predominantly cystocele (63.8%). Regarding intervention protocols, 53.3% underwent combined IncontiLase^®®^ + IntimaLase^®®^ treatment, 35.6% received IntimaLase^®®^ alone, and 11.1% received IncontiLase^®®^ alone. The number of treatment sessions varied based on clinical progression: 48.9% completed two sessions, 40.4% received three sessions, and 10.6% received a single session. Among participants who received only one session, three withdrew from the study, and two discontinued treatment following symptom resolution. Given variability in treatment protocols and number of sessions, no subgroup analyses were performed; this heterogeneity may influence outcome interpretation.

### 3.2. Laser Energy Distribution and Vaginal Anatomical Outcomes

Energy delivered to the anterior vaginal wall varied by session (Session 1: 3462.61 ± 1011.8 J; Session 2: 3200.78 ± 1198.14 J; Session 3: 3943.75 ± 1272.63 J). Posterior wall applications were minimal in Sessions 1 and 2 and absent in Session 3. The 360° mode consistently received the highest energy (e.g., 13,942.28 ± 7592.14 J in Session 1). Vulvar energy decreased progressively across sessions (1104.06 ± 313.71 J to 850.18 ± 240.48 J), consistent with reported symptom improvement. Supplementary Table S1 outlines the distribution of laser pulse energy across anatomical sites and treatment sessions. The consistency of these findings was supported by effect size estimates and corresponding measures of precision.

Vaginal hiatus was significantly reduced at both follow-up assessments (Table 2), with large effect sizes in the comparisons between baseline and FU1 (*r* = 0.99) and between baseline and FU2 (*r* = 0.99), whereas no additional change was observed between FU1 and FU2 (*p* = 0.180), suggesting stabilization of the effect after the first follow-up. Vaginal length showed a slight increase over time; however, differences were non-significant at FU1 (*p* = 0.102) or FU2 (*p* = 0.066), with no difference between follow-up time points (*p* = 0.157). Although trends suggested potential elongation, results did not reach significance. All measurements were performed by the same trained pelvic floor physiotherapist, minimizing inter-rater variability.

### 3.3. Sexual Function, Incontinence Severity, and Quality of Life Outcomes

FSFI total scores remained stable across timepoints (Table 3), with no statistically significant differences and only small to trivial effect sizes across comparisons. Arousal showed a transient reduction at FU1 compared with baseline (*p* = 0.043, *r* = 0.35), corresponding to a moderate effect size. This temporary decline may reflect short-term post-procedural tissue changes (e.g., mild inflammation or sensitivity) and/or behavioral or cognitive factors, such as temporary sexual abstinence or anticipatory discomfort [16,17]. Pain did not differ significantly between baseline and follow-up; however, a significant difference was observed between FU1 and FU2 (*p* = 0.039, *r* = 0.69), indicating a large effect size. No differences were found in desire, lubrication, orgasm or satisfaction over time.

Total ICIQ-SF scores (Table 3) showed no significant differences across timepoints and were accompanied by small to moderate effect sizes. Likewise, severity categories remained broadly stable: mild incontinence remained predominant (baseline: 63.83%; first follow-up: 64.86%; final: 53.33%), moderate incontinence increased slightly at FU2 (40.00%), and severe cases remained rare (<7%). No category-level differences reached significance (*p* > 0.4).

Short-term improvements were observed in I-QOL domains at FU1 although these changes were not sustained at FU2 (Table 3).

### 3.4. Pelvic Floor Strength and Prolapse Outcomes

Table 2 presents the evolution of pelvic floor muscle strength and prolapse staging across compartments. Pelvic floor muscle strength, assessed with the Oxford Scale, showed no significant change across evaluations (*p* > 0.3). Most participants remained in the weak (2–3) or flicker (1) categories throughout.

Cystocele staging improved significantly, with the proportion of women without anterior compartment prolapse increasing from 36.17% at baseline to 38.30% at FU2, and stage III cases decreasing from 12.77% to 6.38% (*p* = 0.013 for baseline vs. FU2; *p* = 0.046 for FU1 vs. FU2). Uterine prolapse and rectocele showed no statistically significant changes. Staging assessments were not blinded, as they were performed in the course of routine clinical care; however, all examinations were conducted by the same clinician using standardized POP-Q procedures to minimize inter-rater variability and enhance measurement consistency.

#### Correlation Between Laser Energy and Clinical Outcomes

Correlations between laser energy and clinical outcomes are presented in Supplementary Table S2.

Several significant associations emerged, although most variables showed no correlation. Key findings:

- Higher vulvar energy at Session 2 correlated with lower ICIQ-SF scores (r = −0.424; *p* = 0.016).
- Higher 360° energy in Session 1 correlated with greater vaginal length (r = 0.362; *p* = 0.024).
- Higher anterior wall energy in Session1 correlated with presence of uterine prolapse (r = 0.433; *p* = 0.007).
- No significant correlations were observed with FSFI total scores, I-QOL total scores, rectocele, or pelvic floor strength.

These findings should be considered exploratory and hypothesis-generating only, and should not be interpreted as evidence of dose–response relationships.

## 4. Discussion

The present study aimed to evaluate changes in vaginal hiatus, vaginal length, urinary incontinence severity, pelvic floor muscle strength, quality of life, and female sexual function following treatment with non-ablative Er:YAG vaginal laser therapy (IntimaLase^®®^ and IncontiLase^®®^) in postmenopausal women with GSM through serial assessment. The findings suggest that non-ablative Er:YAG laser therapy may be associated with modest anatomical changes and short-term improvements in selected quality of life domains, although effects were inconsistent across functional outcomes.

A significant reduction in vaginal hiatus was observed at both follow-up time points, indicating a sustained contraction of the vaginal introitus following treatment. This aligns with the proposed mechanism of Er:YAG lasers, which are hypothesized to induce collagen remodeling, neocollagenesis, and reorganization of elastic fibers, potentially contributing to connective tissue tightening [5,6], although these mechanisms have not been directly confirmed in relation to clinical outcomes in this study. The reduction in cystocele severity observed at FU2 is consistent with previously reported improvements in anterior compartment support [18].

Although vaginal length increased slightly after treatment, these changes did not reach statistical significance. However, the positive correlation between 360° energy delivery and vaginal length suggests that circumferential thermal stimulation may influence vaginal tissue elasticity. Similar observations have been reported in studies demonstrating laser-associated epithelial regeneration and increased mucosal elasticity [8,19].

Sexual function, assessed through the FSFI, demonstrated overall stability, with no significant changes in the total score or in most individual domains. The transient reduction in arousal observed after the first session may be related to acute inflammatory changes or local sensitivity following laser exposure. Contrary to several studies [7,20] the present study did not identify significant improvements in pain or lubrication domains. This lack of improvement may also reflect the short follow-up period and the multifactorial nature of sexual function, which may not respond to isolated structural changes. Differences in treatment frequency, device parameters, or baseline severity may account for these discrepancies. The absence of notable sexual function improvement may also reflect the complex, multifactorial nature of sexual health, which is influenced by hormonal status, psychological well-being, relationship factors, and sexual activity frequency, variables not controlled in this study [21].

Urinary incontinence severity, measured through the ICIQ-SF, did not demonstrate significant changes at the group level. These findings differ from several reports suggesting symptom improvement following non-ablative laser therapy, particularly among women with mild or moderate stress urinary incontinence [11,22]. Other studies noted that the laser’s effect on continence could be related to the contraction of periurethral tissue and the improvement of the closure mechanism, although this effect does not always translate into significant changes in general questionnaires [7]. A meta-analysis suggested that, although vaginal laser shows potential benefits in urinary incontinence, the methodological heterogeneity of the studies limits the strength of the conclusions [12]. Additionally, the absence of a control group limits the ability to determine whether observed patterns differ from natural variability or placebo effects.

Quality of life measures showed short-term improvements in psychosocial impact, social embarrassment, and behavioral limitations at FU1; however, none of these improvements were sustained at FU2. These transient improvements may reflect early subjective perception of symptom relief, a phenomenon also described in other intervention studies on urinary incontinence [23,24]. Placebo and expectation effects are well documented in studies involving energy-based interventions and subjective outcomes. Sham-controlled trials of vaginal laser therapies have demonstrated that part of the observed benefit may be attributable to non-specific effects rather than direct physiological mechanisms. The rapid decline of improvements between FU1 and FU2 highlights the need to assess the durability of laser effects over longer periods. Placebo effects, heightened expectations, or non-specific treatment effects may also contribute to early positive responses, especially in uncontrolled studies [10]. Therefore, these short-term improvements should be interpreted cautiously.

The short follow-up duration represents an additional limitation. While collagen remodeling and tissue regeneration processes may evolve over several months, early post-treatment assessments primarily capture initial responses rather than sustained effects. Future studies should incorporate longer follow-up periods (e.g., ≥6–12 months) to better evaluate durability.

These findings are relevant for primary care and women’s health services, where GSM is frequently under-recognized and managed longitudinally, highlighting the need for accessible non-hormonal options within routine outpatient care pathways.

Pelvic floor muscle strength remained unchanged across evaluations, reinforcing that non-ablative laser therapy primarily targets connective tissue rather than muscular structures. This is consistent with prior evidence indicating that laser therapy does not replicate the effects of active pelvic floor muscle training [10]. The trend toward applying higher anterior wall energy in women with uterine prolapse may reflect clinician adaptation to baseline severity but does not imply therapeutic effect on apical prolapse, which is consistent with other studies that identified greater laser effectiveness in the anterior compartment [12]. Some studies propose that combining laser therapy with pelvic floor physiotherapy programs could enhance the results, opening a relevant avenue for future research highlighting the importance of multimodal approaches rather than isolated structural interventions [21].

The inconsistency between significant anatomical improvements (e.g., reduced vaginal hiatus, improved cystocele stage) and limited functional or subjective improvements (e.g., sexual function, urinary incontinence, I-QOL) underscores the multifactorial nature of GSM. This discrepancy may reflect the distinction between structural tissue remodeling and neuromuscular or perceptual function. Functional outcomes such as continence and sexual function depend on complex interactions including neuromuscular coordination, behavioral adaptation, and psychological factors, which are not directly addressed by tissue-level interventions. Anatomical or connective tissue changes do not necessarily translate into perceivable functional capacity or improved sexual response. These discrepancies may also reflect insufficient follow-up duration, as symptom improvements in laser therapy may require cumulative tissue remodeling over several months. Similar dissociations between anatomical findings (e.g., POP-Q staging) and symptom severity have been widely reported in pelvic floor literature [25,26]. Additionally, these design features increase susceptibility to non-specific treatment effects and limit internal validity.

The absence of a control group is a major methodological limitation. Without a comparator such as sham treatment or standard care, it is not possible to distinguish treatment-related effects from placebo responses, regression to the mean, or natural symptom fluctuation. This is particularly relevant for subjective outcomes, where expectation bias may play a significant role. This study has several important limitations. The single-arm design without a control group precludes causal inference and increases susceptibility to placebo and expectation bias, regression to the mean, and natural symptom variability. The short follow-up period limits the assessment of durability, as tissue remodeling processes may evolve over longer periods. Treatment heterogeneity, including variability in protocols and the number of sessions, reduces reproducibility and may influence treatment response. Manual anatomical measurement may introduce measurement bias, and the lack of full assessor blinding introduces potential observer bias, which may have influenced outcome assessment. Additionally, the absence of adjustment for multiple comparisons and the fact that the study was not powered for multiple endpoints increase the risk of Type I error. Although effect sizes were reported to support interpretation, the precision of estimates remains limited. Baseline heterogeneity, including variability in symptom severity and sexual activity, may also have influenced outcomes.

Despite these limitations, the study provides integrated anatomical and patient-reported outcomes within a real-world care context, an approach directly relevant to chronic care pathways for GSM. These findings are preliminary and support future randomized studies with longer follow-up and comprehensive patient-reported outcomes to establish durability and clinical relevance.

From an implementation perspective, the results may inform the design of pragmatic trials and outpatient care pathways for non-hormonal, minimally invasive options for GSM within primary care and women’s health services.

## 5. Conclusions

This preliminary single-arm study indicates that non-ablative Er:YAG vaginal laser therapy, using the IntimaLase^®®^ and IncontiLase^®®^ protocols, was well tolerated and may be associated with modest anatomical benefits such as reduced vaginal hiatus and modest improvements in cystocele severity. Early improvements in select domains of quality of life were also observed but were not sustained at later follow-up. Functional outcomes, including urinary incontinence, pelvic floor muscle strength, and sexual function, showed limited or inconsistent improvements. These findings should be interpreted cautiously given the methodological limitations and require confirmation in well-designed randomized controlled trials.

Although exploratory correlations indicate that certain energy delivery parameters may influence specific outcomes, these associations cannot establish causality and require confirmation in controlled settings. Given the methodological limitations, including the absence of a control group, short-term follow-up, manual anatomical measurements, and small sample size, future randomized controlled trials with longer and standardized follow-up periods, objective imaging tools, and stratified patient phenotypes are essential to fully define the true clinical impact, durability, and therapeutic role of non-ablative Er:YAG laser therapy in GSM.

## Figures and Tables

**Table 1:** Baseline clinical and gynecologic characteristics of postmenopausal women (*n* = 47).

Characteristic	Value
Age (years), mean ± SD	58.11 ± 8.99
Body mass index (kg/m^2^), mean ± SD	23.21 ± 3.64
Full-term births, *n* (%)	
1	11 (25.00)
2	23 (52.27)
3	7 (15.91)
Preterm births, *n* (%)	1 (2.27)
Urinary incontinence, *n* (%)	
Yes	22 (46.81)
No	25 (53.19)
Pelvic organ prolapse, *n* (%)	
Yes	31 (65.96)
No	16 (34.04)
Type of prolapse, *n* (%) *	
Cystocele	30 (63.83)
Uterine prolapse	10 (21.28)
Rectocele	27 (57.45)
Laser protocol, *n* (%)	
IncontiLase^®®^	5 (11.11)
IntimaLase^®®^	16 (35.56)
Both (IncontiLase^®®^ + IntimaLase^®®^)	24 (53.33)
Number of treatment sessions, *n* (%)	
One	5 (10.64)
Two	23 (48.94)
Three	19 (40.43)

* Percentages for prolapse types may exceed 100% because categories are not mutually exclusive. SD: standard deviation.

**Table 2:** Changes in anatomical parameters, pelvic floor muscle strength, and pelvic organ prolapse staging after non-ablative Er:YAG laser treatment (*n* = 47).

Outcome	Timepoint	Median	p25 or *n*	p75 or %	*p* (EB vs. FU1)	*p* (EB vs. FU2)	*p* (FU1 vs. FU2)
Vaginal hiatus (cm)	EB	2.5	2.0	3.0	<0.001 *r* = 0.990	<0.001 *r* = 0.990	0.180 *r* = 1.000
	FU1	2.0	1.0	3.0			
	FU2	2.0	1.0	3.0			
Vaginal length (cm)	EB	8.5	7.3	9.0	0.102 *r* = 1.00	0.066 *r* = 1.00	0.157 *r* = 1.00
	FU1	8.5	8.0	9.0			
	FU2	9.0	8.0	9.0			
Oxford scale (pelvic floor muscle strength), *n* (%)	EB	None	7	20.00	1.000 *r* = NA	0.317 *r* = 1.00	0.317 *r* = 1.00
		Flicker	10	28.57			
		Weak	16	45.71			
		Moderate	2	5.71			
	FU1	None	7	20.00			
		Flicker	10	28.57			
		Weak	16	45.71			
		Moderate	2	5.71			
	FU2	None	8	22.86			
		Flicker	9	25.71			
		Weak	16	45.71			
		Moderate	2	5.71			
Cystocele stage, *n* (%)	EB	None	17	36.17	0.059 *r* = 0.71	0.013 *r* = 0.80	0.046 *r* = 1.00
		Stage I	9	19.15			
		Stage II	15	31.91			
		Stage III	6	12.77			
	FU1	None	17	36.17			
		Stage I	12	25.53			
		Stage II	14	29.79			
		Stage III	4	8.51			
	FU2	None	18	38.30			
		Stage I	13	27.66			
		Stage II	13	27.66			
		Stage III	3	6.38			
Uterine prolapse stage, *n* (%)	EB	None	37	78.72	0.317 *r* = 1.00	0.317 *r* = 1.00	1.000 *r* = NA
		Stage I	6	12.77			
		Stage II	4	8.51			
	FU1	None	38	80.85			
		Stage I	5	10.64			
		Stage II	4	8.51			
	FU2	None	38	80.85			
		Stage I	5	10.64			
		Stage II	4	8.51			
Rectocele stage, *n* (%)	EB	None	20	42.55	0.102 *r* = 0.67	0.058 *r* = 0.72	0.157 *r* = 1.00
		Stage I	18	38.30			
		Stage II	8	17.02			
		Stage III	1	2.13			
	FU1	None	20	42.55			
		Stage I	22	46.81			
		Stage II	4	8.51			
		Stage III	1	2.13			
	FU2	None	22	46.81			
		Stage I	20	42.55			
		Stage II	4	8.51			
		Stage III	1	2.13			

EB: baseline (pre-treatment evaluation); FU1: first follow-up; FU2: second follow-up. *p*-values derived from (two-tailed) Wilcoxon signed-rank tests for paired comparisons. Effect size was estimated as r = |Z|/√N, where N corresponds to the number of non-zero paired differences. NA: not estimable because there were no non-zero paired differences.

**Table 3:** Questionnaire scores on sexual function, urinary incontinence, and incontinence-specific quality of life in postmenopausal women (*n* = 47).

Instrument/Domain	Timepoint	Median	p25	p75	*p* (EB vs. FU1)	*p* (EB vs. FU2)	*p* (FU1 vs. FU2)
FSFI total score	EB	18.70	15.20	21.00	0.353 *r* = 0.163	0.836 *r* = 0.065	0.875 *r* = 0.061
	FU1	18.20	14.60	20.20			
	FU2	18.40	16.55	20.80			
Desire	EB	3.00	2.40	4.20	0.519 *r* = 0.230	0.265 *r* = 0.287	0.172 *r* = 0.400
	FU1	3.60	2.40	4.20			
	FU2	3.00	2.40	4.20			
Arousal	EB	3.30	2.70	4.20	0.020 *r* = 0.346	0.145 *r* = 0.298	0.495 *r* = 0.364
	FU1	2.40	1.50	3.90			
	FU2	2.90	1.80	3.90			
Lubrication	EB	3.60	3.00	3.60	0.736 *r* = 0.002	0.218 *r* = 0.408	0.865 *r* = 0.129
	FU1	3.60	1.20	3.90			
	FU2	3.50	3.20	3.80			
Orgasm	EB	3.20	2.80	4.00	0.109 *r* = 0.312	0.297 *r* = 0.312	0.588 *r* = 0.229
	FU1	2.80	2.40	4.00			
	FU2	3.00	2.60	3.60			
Satisfaction	EB	2.00	1.20	2.80	0.233 *r* = 0.302	0.950 *r* = 0.062	0.512 *r* = 0.301
	FU1	1.60	0.80	2.40			
	FU2	1.60	1.20	3.00			
Pain	EB	4.00	1.20	5.20	0.216 *r* = 0.210	0.229 *r* = 0.223	0.084 *r* = 0.689
	FU1	3.60	0.40	4.80			
	FU2	4.40	2.80	5.80			
I-QOL total score	EB	34.0	28.0	48.0	0.308 *r* = 0.170	0.402 *r* = 0.268	0.694 *r* = 0.132
	FU1	33.0	26.0	40.0			
	FU2	34.0	27.0	52.0			
Behavioral limitations	EB	13.0	10.0	17.0	0.013 *r* = 0.389	0.721 *r* = 0.156	0.726 *r* = 0.127
	FU1	11.0	8.0	16.0			
	FU2	13.0	11.0	22.0			
Psychosocial impact	EB	14.0	10.0	19.0	0.002 *r* = 0.489	0.329 *r* = 0.267	0.539 *r* = 0.202
	FU1	10.0	8.0	14.0			
	FU2	12.0	10.0	20.0			
Social embarrassment	EB	7.0	6.0	9.0	0.002 *r* = 0.550	0.593 *r* = 0.723	0.721 *r* = 0.118
	FU1	6.0	5.0	8.0			
	FU2	7.0	6.0	13.0			
ICIQ-SF total score	EB	4.0	1.0	8.0	0.108 *r* = 0.237	0.210 *r* = 0.384	0.798 *r* = 0.050
	FU1	3.0	1.0	8.0			
	FU2	5.0	1.0	11.0			

Severity of urinary incontinence remained predominantly mild across time (baseline: 63.83%, FU1: 64.86%, FU2: 53.33%), with no significant changes in category distribution (all *p* > 0.4). FSFI: Female Sexual Function Index; ICIQ-SF: International Consultation on Incontinence Questionnaire—Short Form; I-QOL: Incontinence Quality of Life Questionnaire. *p*-values derived from Wilcoxon signed-rank tests for paired comparisons. Effect size was estimated as r = |Z|/√N, where N corresponds to the number of non-zero paired differences.

## Data Availability

Available from the corresponding author on reasonable request. The data are not publicly available due to privacy restrictions.

## Supplementary Materials

The following supporting information can be downloaded at https://www.mdpi.com/article/10.3390/healthcare14091180/s1: Table S1: Laser energy distribution across anatomical regions and treatment sessions; Table S2: Correlations between laser energy delivery and clinical outcomes.

## Abbreviations

The following abbreviations are used in this manuscript:

BE	Baseline Evaluation
BMI	Body Mass Index
FSFI	Female Sexual Function Index
FU1	First Follow-up
FU2	Second Follow-up
GSM	Genitourinary syndrome of menopause
ICIQ-SF	International Consultation on Incontinence Questionnaire—Short Form
I-QOL	Incontinence Quality of Life Questionnaire
PFD	Pelvic Floor Dysfunction
POP-Q	Pelvic Organ Prolapse Quantification
SD	Standard Deviation
SUI	Stress Urinary Incontinence
