# Supplementary material for: Anatomical and Patient-Reported Outcomes After Non-Ablative Er:YAG Laser Therapy for Genitourinary Syndrome of Menopause: A Prospective Real-World Cohort Study
Source: Healthcare (Basel). 2026 Apr 28;14(9):1180. doi: 10.3390/healthcare14091180 (PMC13163903; doi:10.3390/healthcare14091180)
Supplement: Supplementary file 1 [file healthcare-14-01180-s001.zip › healthcare-4189623-supplementary.pdf]

Supplementary Table S1. Laser energy distribution across anatomical regions and treatment sessions.

Region	Session 1 (J)	Session 2 (J)	Session 3 (J)	Description
Anterior vaginal wall	3462.61 ± 1011.80	3200.78 ± 1298.14	3943.75 ± 1272.63	Highest energy in Sessions 1–3 among targeted wall applications.
Posterior vaginal wall	Minimal	Minimal	Not applied	Posterior wall treated minimally or omitted based on clinical assessment.
360° circumferential mode	13,942.28 ± 7592.14	12,236.53 ± 6730.11	14,105.31 ± 8321.98	Mode receiving the highest overall energy delivery.
Vulvar region	1104.06 ± 313.71	971.15 ± 285.38	850.18 ± 240.48	Energy decreased progressively as symptoms improved.

Supplementary Table S2. Correlations between laser energy delivery and clinical outcomes.

Laser Energy Parameter	Outcome	Correlation (r)	p-value
360° energy (Session 1)	Vaginal length (FU1)	0.362	0.024
Vulvar energy (Session 2)	ICIQ-SF total score (FU2)	-0.424	0.016
Anterior wall energy (Session 1)	Presence of uterine prolapse	0.433	0.007
